# Carbon Storage Distribution Characteristics of Vineyard Ecosystems in Hongsibu, Ningxia

**DOI:** 10.3390/plants10061199

**Published:** 2021-06-11

**Authors:** Liang Zhang, Tingting Xue, Feifei Gao, Ruteng Wei, Zhilei Wang, Hua Li, Hua Wang

**Affiliations:** 1 College of Enology, Northwest A&F University, Yangling 712100, China; zhangliang20@nwafu.edu.cn (L.Z.); gaofeifei@nwafu.edu.cn (F.G.); weiruteng2019@nwafu.edu.cn (R.W.); wangzhilei@nwafu.edu.cn (Z.W.); 2 School of Food & Wine, Ningxia University, Yinchuan 750021, China; xuetingting@nwafu.edu.cn; 3 Shaanxi Engineering Research Center for Viti-Viniculture, Yangling 712100, China; 4 China Wine Industry Technology Institute, Yinchuan 750021, China

**Keywords:** winegrape, vineyard ecosystems, allometric, carbon storage, grapevines biomass, carbon distribution

## Abstract

Given that the global winegrape planting area is 7.2 × 10^6^ hm^2^, the potential for winegrape crop-mediated carbon capture and storage as an approach to reducing greenhouse gas emissions warranted further research. Herein, we employed an allometric model of various winegrape organs to assess biomass distributions, and we evaluated the carbon storage distribution characteristics associated with vineyard ecosystems in the Hongsibu District of Ningxia. We found that the total carbon storage of the *Vitis vinifera* ‘Cabernet Sauvignon’ vineyard ecosystem was 55.35 t·hm^−2^, of which 43.12 t·hm^−2^ came from the soil, while the remaining 12.23 t·hm^−2^ was attributable to various vine components including leaves (1.85 t·hm^−2^), fruit (2.16 t·hm^−2^), canes (1.83 t·hm^−2^), perennial branches (2.62 t·hm^−2^), and roots (3.78 t·hm^−2^). Together, these results suggested that vineyards can serve as an effective carbon sink, with the majority of carbon being sequestered at the soil surface. Within the grapevines themselves, most carbon was stored in perennial organs including perennial branches and roots. Allometric equations based on simple and practical biomass and biometric measurements offer a means whereby grape-growers and government entities responsible for ecological management can better understand carbon distribution patterns associated with vineyards.

## 1. Introduction

Over the past century, the global climate has undergone persistent warming associated with a rise in atmospheric CO_2_ content, which is considered to be the most important driver of such global warming [1,2]. Efforts to combat such climate change have consisted of studies of energy conservation and emission reduction, carbon sources, carbon sinks, and carbon sequestration in agricultural ecosystems [3,4,5]. Orchards represent an important facet of the overall agricultural ecosystem, and exhibit a greater amount of carbon fixation per unit area relative to grasslands [6,7], while being easier to control than non-economic forest ecosystems. A number of interventional strategies such as rational fertilization, cover crops, non-tillage, and other agronomic measures can enhance soil structure and associated productivity, thereby improving efficiency within the orchard ecosystem while simultaneously reducing net carbon emissions.

Winegrapes are widely cultivated in China, where they represent an important cash crop, facet of the agricultural economy, and an important part of the orchard ecosystem. Hongsibu is located at the eastern foot of Helan Mountain in the Ningxia province of Northwest China, which is a major wine-producing region in China and one of several with nationally protected geographical designations. Grapevines are representative of the economic forestry industry in Hongsibu, and can store carbon for extended periods [8]. One study found that Cabernet Sauvignon vineyards store carbon at a rate of 42.75 t·hm^−2^, while for Chardonnay vineyards this carbon storage levels were roughly 8.02 t·hm^−2^ [9]. The total winegrape cultivation area in Honsibu is approximately 7067 hm^2^, with an annual production of 9100 tons of wine and an economic value of 350 million RMB in 2018.

While many environmental and economic aspects of viticulture are the subject of active research, there is growing scientific interest in the relationship between carbon emissions and vineyard management [10,11,12]. Specifically, the widespread and perennial nature of vineyard ecosystems has led to rising interest from both the public sector and growers in reducing viticulture-related greenhouse gas emissions. Internationally, the wine industry exhibits a growing focus on carbon accounting protocols that underscore the interest of consumers and the industry as a whole in minimizing or offsetting carbon emissions associated with winegrape production. The development of a straightforward, wine production-specific approach to measuring vineyard and winery carbon emissions would thus offer scalable benefits [13]. At present, however, the carbon storage and emission characteristics of viticultural activities remain poorly understood, particularly in cases where time-intensive empirical studies are required [14]. Relatively little field-derived data pertaining to factors including vine biomass, cover crop biomass, and soil carbon storage capacity is available, limiting more robust carbon accounting within this wine sector [15].

A growing number of studies in recent years have explored carbon storage within vineyard ecosystems [16,17,18]. An allometric model has been employed to successfully study carbon levels within vineyards, revealing that grapevines achieved an average carbon storage level of 12.3 t·hm^−2^, of which perennial biomass accounted for 8.9 t·hm^−2^, whereas annual leaves and canes accounted for just 1.7 t·hm^−2^, and fruit accounted for another 1.7 t·hm^−2^ [19]. Levels of carbon stored in a Merlot vineyard ranged from 5.72 (±0.07)–7.23 (±1.11) t C ha^−1^ year^−1^ based upon net ecosystem production (NEP), suggesting that properly managed vineyards have the potential to serve as carbon sinks [20]. Monitoring of the net ecosystem CO_2_ exchange (NEE) of a commercial vineyard in northeastern Italy confirmed the potential of a vineyard ecosystem to serve as a net carbon sink, with absorption of approximately −233 g C m^−2^ [21]. In China, researchers have found that young and mature citrus orchard ecosystems are associated with carbon storage levels of 157.90 t·hm^−2^ and 214.63 t·hm^−2^, respectively, with soil accounting for over 70% of this storage [22]. Studies of such soil carbon storage have the greatest impact on understanding the overall carbon levels stored within vineyard ecosystems [23,24,25,26], and the soil organic carbon fractions, including soil microbial biomass carbon (MBC) and dissolved organic carbon (DOC), are among of the most important factors linked with such carbon storage [27,28,29].

The present study was designed with three primary objectives: (1) to conduct a field sampling-based assessment of major biomass fractions (i.e., roots, perennial branches, canes, leaves, and fruit) in order to measure standing vine biomass and thereby determine the carbon storage characteristics of vineyards; (2) to establish an approach to measuring the carbon storage distribution characteristics of vineyard ecosystems based upon readily measured physical vine properties such as trunk diameter, pruning weight, or fruit weight; and (3) to define allometric relationships that will enable growers and land managers to quickly assess vineyard carbon stocks.

## 2. Results

### 2.1. Establishment of an Allometric Model of Grapevine Biomass

As discussed in the Methods section of this study, an allometric model (Y = aX^b^) incorporating standard vine trunk base diameter and organ biomass measurements was established. This model enables the calculation of the total biomass of each organ per unit area, based upon planting density. The trunk base diameter was significantly correlated with the biomass of each organ for these four grape cultivars, confirming that trunk base diameter can be measured in order to accurately estimate the biomass of each organ associated with these vines (Table 1).

### 2.2. Biomass Distribution in Vines and Vineyards

**Figure 1:**
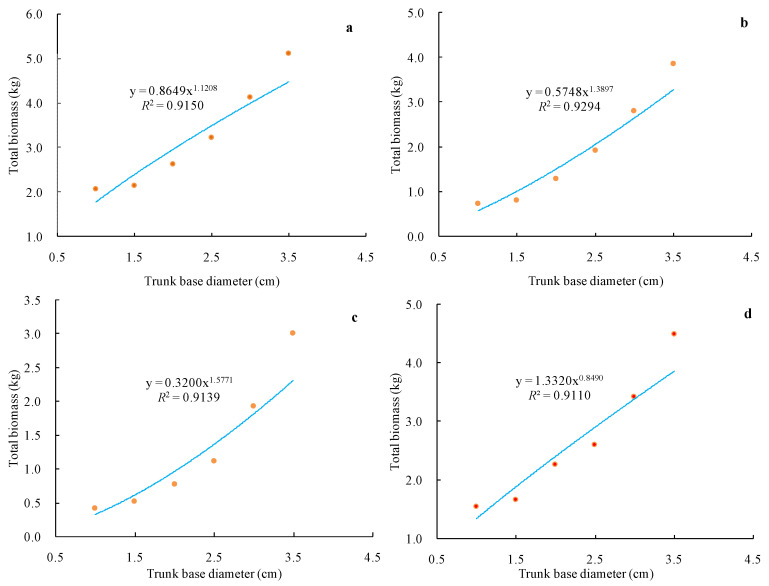
Changes of total vine biomass in different trunk base diameter classes. (**a**) Cabernet Sauvignon, (**b**) Merlot, (**c**) Chardonnay, (**d**) Italian Riesling.

After a regression model of the relationship between vine biomass and trunk base diameter was established, total vine biomass was found to be significantly correlated with trunk base diameter, and vine biomass increased with rising diameter class (Figure 1). For Cabernet Sauvignon samples, the regression equation for trunk base diameter and total biomass was Y = 0.8649 X^1.1208^ (*R*^2^ = 0.9150 **), with this correlation being highly significant. Trunk base diameters ranged from 0.75–3.8 cm, with biomass per vine ranging from 2.06–5.11 kg. The total biomass values for red winegrape varieties were higher than those of age-matched white winegrape varieties, and the individual biomass values for each red winegrape organ were higher than those of the corresponding white winegrape organs.

**Figure 2:**
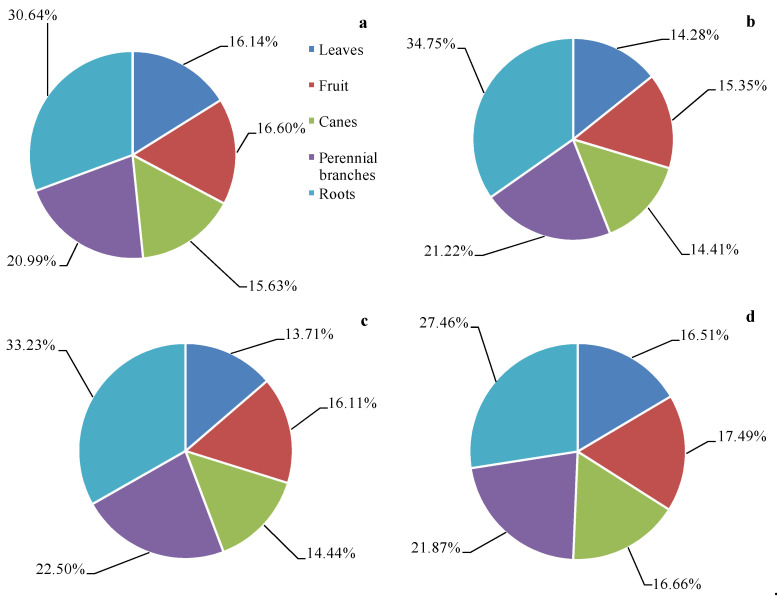
Percentage of total biomass of each organ per unit area. (**a**) Cabernet Sauvignon, (**b**) Merlot, (**c**) Chardonnay, (**d**) Italian Riesling.

After calculating the total biomass of each organ per unit area, the proportion of the total biomass of each organ per unit area was found to differ significantly (Figure 2). In the four analyzed vineyards, the proportion of total biomass associated with each organ was, in order, roots > perennial branches > fruits > canes > leaves, with the exception of Cabernet Sauvignon vineyards. In the Merlot vineyards, the root total biomass per unit area accounted for 34.75% of the overall biomass, whereas leaves accounted for just 14.28% of overall vine biomass. Additionally, the total biomass of red varieties per unit area was higher than that of age-matched white varieties, with the biomass of all organs of red varieties per unit area being higher than the corresponding values for the white varieties.

### 2.3. Carbon Storage and Distribution Characteristics in Vineyard Ecosystems

#### 2.3.1. Carbon Storage and Distribution Characteristics in Grapevine Biomass

For analyzed standard vines, carbon levels in each organ rose with trunk base diameter, and there were also differences in the carbon content of different organs within a given diameter class (Table 2). The average carbon content was highest in the fruit (all > 450 g/kg), followed by the perennial branches, roots, canes, and leaves. Within a given diameter class, the fruit carbon content of Cabernet Sauvignon and Italian Riesling grapes was significantly higher than that of other analyzed organs (*p* < 0.05).

Carbon storage in grapevine biomass is dependent upon a number of factors, including planting density, biomass, and carbon content. With respect to carbon stock distributions in various organs collected from Merlot grapevines, root carbon storage was equal to 1.54 t·hm^−2^, accounting for 34.87% of the total carbon storage, while the carbon storage of perennial branches was 0.96 t·hm^−2^ (21.67%). Carbon storage density for fruit and canes accounted for 16.29% and 13.79% of the total carbon storage, respectively, while leaves accounted for just 13.39% of the total carbon storage (see Supplementary Materials, Table S1). The carbon storage distribution characteristics of Chardonnay and Italian Riesling were consistent with those of the red winegrape varieties (roots > perennial branches > fruit > canes > leaves). Together, these results suggest that perennial organs (roots and perennial branches) are the primary sources of carbon storage within the overall grapevine biomass.

#### 2.3.2. Carbon Storage and Distribution Characteristics in Soil

Soil carbon storage in vineyards is dependent upon factors including the level of soil organic carbon content and soil bulk density. Herein, we assessed soil carbon levels and soil bulk density at four depth levels in analyzed vineyards, revealing a pronounced vertical distribution pattern for both of these parameters, such that the highest soil organic carbon level and the lower soil bulk density were present in the 0–20 cm soil layer (Table 3).

The carbon storage of each soil layer decreased with increasing soil depth, with carbon storage being primarily concentrated in the surface soil (0–20 cm). In Cabernet Sauvignon vineyards, carbon storage was equal to 26.38 t·hm^−2^ in the 0–40 cm layer, accounting for 61.19% of total soil carbon storage. The topsoil (0–20 cm) exhibited the highest soil carbon storage (15.03 t·hm^−2^), accounting for 34.87% of overall carbon storage. In the Chardonnay vineyard, the total carbon storage was 11.86 t·hm^−2^ in the 0–20 cm layer, accounting for 32.31% of the total carbon storage (Table 3).

#### 2.3.3. Carbon Storage and Distribution Characteristics in Vineyard Ecosystems

In the Cabernet Sauvignon vineyard, the total carbon storage was equal to 55.35 t·hm^−2^, of which the biomass carbon storage was just 12.23 t·hm^−2^, accounting for 22.10% of the total carbon storage in the vineyard ecosystem. In contrast, soil carbon storage amounted to 43.12 t·hm^−2^, accounting for 77.90% of the total carbon storage (Table 4). This indicates the soil is the primary site of carbon storage in vineyards, with vines playing a relatively small role in such storage. Relative to similarly aged white vineyards, red vineyards exhibited higher levels of carbon storage.

### 2.4. Changes in Rhizosphere Soil MBC and DOC

**Figure 3:**
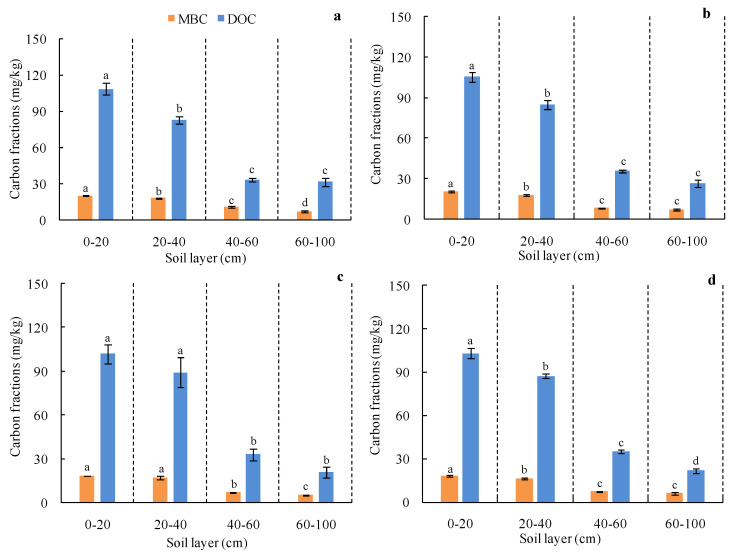
Vertical distributions of MBC and DOC in the rhizosphere soil. (**a**) Cabernet Sauvignon, (**b**) Merlot, (**c**) Chardonnay, (**d**) Italian Riesling.

MBC and DOC contents gradually decreased as soil depth increased (Figure 3). These soil MBC and DOC levels were highest in the 0–20 cm Cabernet Sauvignon rhizosphere soil layer (20.33 mg/kg and 108.58 mg/kg, respectively) (Figure 3a). In vineyards growing red winegrape cultivars, MBC and DOC in the 0–20 cm layer were significantly higher than in other soil layers, and the MBC and DOC in the 20–40 cm layer were also significantly higher than in the 40–60 cm and 60–100 cm layers (*p* < 0.05). In the Chardonnay vineyard, the MBC and DOC in the 0–40 cm layer were significantly higher than that in the 40–60 cm and 60–100 cm layers (*p* < 0.05) (Figure 3c). Overall, these results suggest that soil MBC and DOC are mainly concentrated in the topsoil (0–20 cm, 20–40 cm).

### 2.5. Correlation Analyses

**Figure 4:**
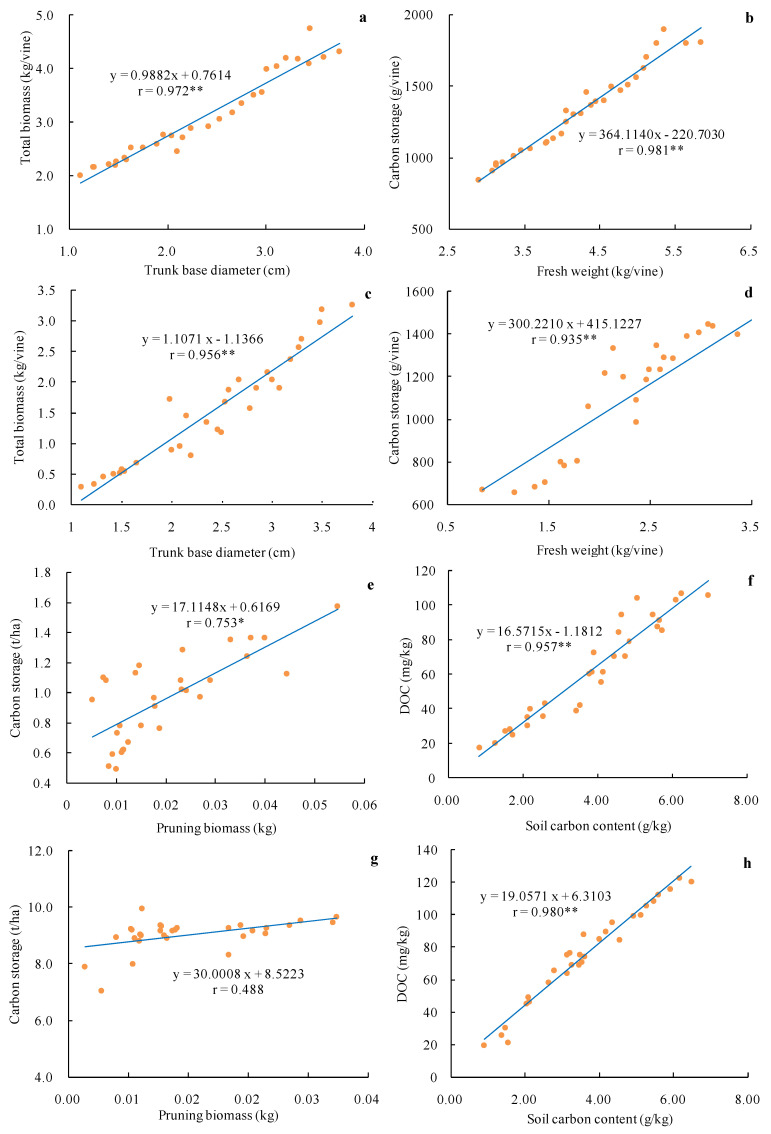
Correlation analysis of vineyard characteristics. (**a**,**b**) Cabernet Sauvignon, (**c**,**d**) Merlot, (**e**,**f**) Chardonnay, (**g**,**h**) Italian Riesling. * *p* < 0.05; ** *p* < 0.01. *n* = 30.

Correlation analyses revealed that the trunk base diameter and total biomass of the vine were significantly correlated in the Cabernet Sauvignon (Figure 4a,b) and Merlot (Figure 4c,d) vineyards, as were fresh weight and vine carbon storage. In the Chardonnay vineyards, pruning weight was significantly correlated with total carbon storage (Figure 4e), while in the Italian Riesling vineyards, these two variables were unrelated (Figure 4g). For the white grape varieties, the correlations between soil organic carbon and DOC were extremely significant (Figure 4f,h). Correlation analyses indicated that the total vine biomass and organ carbon fractions were the primary factors that determined the overall carbon storage of a given vineyard.

## 3. Discussion

Herein, we established a biomass model (Table 1) and used it to measure spatial carbon storage distributions in storage ecosystems. These analyses revealed that carbon stores in grapevines were primarily located in perennial organs (perennial branches and roots), consistent with the findings of other studies [19]. Total vineyard ecosystem carbon stores are equal to the sum of carbon stored in vines, soil, and ground cover weeds. We did not take the weeds into account, and so our model may underestimate the overall carbon storage capacity of vineyards. We further determined that soil carbon storage in the analyzed vineyards was 3.5–13.3 times that of the grapevines, suggesting that the soil is the primary site of carbon storage within these vineyard ecosystems. This is in line with reports from citrus [30] and mango orchards [31]. We additionally established the carbon content of different grapevine organs (Table 2), measured soil carbon content (Table 3), and examined vine biomass distribution characteristics (Figure 1 and Figure 2).

Overall, our analysis serves as a more accurate and granular assessment of grapevine biomass distributions relative to prior studies. When assessing vineyard biomass characteristics, we separated the vines into multiple diameter classes for the purposes of modeling (Table 1), given that vine sizes were not consistent within or among vineyards. We found that current carbon storage levels in the analyzed Cabernet Sauvignon, Merlot, Chardonnay, and Italian Riesling vineyards were 12.23 t·hm^−2^, 4.41 t·hm^−2^, 2.77 t·hm^−2^, and 9.89 t·hm^−2^, respectively (Table 4), in line with a prior report from California indicating that vine carbon stores ranged from 5.5 to 11.0 Mg C ha^−1^ [32]. Other researchers have assessed vineyard ecosystems and concluded that they store large amounts of carbon [33]. For example, one study of three Chinese vineyards found that the total carbon stores in 5-year-old, 10-year-old, and 18-year-old vineyards were 55.41 t·hm^−2^, 66.92 t·hm^−2^, and 77.04 t·hm^−2^, respectively [34]. These prior carbon store levels were higher than those in the present study, which may be attributable to the relatively young vines we analyzed in this study. Another study of vineyards in northern California measured 3.0 t·hm^−2^ of aboveground carbon storage, whereas soil carbon storage was as high as 84.1 t·hm^−2^ [35]. Their aboveground biomass carbon storage was equivalent to that measured in this study, while their calculated soil carbon storage was substantially higher, potentially because in the present study the analyzed soil was of a sandy loam variety with limited carbon content. However, the present study has only one year of experimental data to illustrate the distribution characteristics of carbon storage in vineyard ecosystems. This conclusion may be merely preliminary, and requires further research in the future.

There have been few analyses to date regarding vineyard belowground biomass and carbon storage. One report studied vineyard carbon stores but did not include root biomass in their calculations [35], even though root systems are estimated to account for 30% of overall vine biomass [36]. Herein, we measured belowground root carbon storage and found such storage to be higher, with root carbon storage for Cabernet Sauvignon, Merlot, Chardonnay, and Italian Riesling varieties accounting for 30.87%, 34.87%, 33.37%, and 27.73% of the total carbon storage for these vines, respectively (Table S1). Brunori et al. (2016) [20] studied the ability of grapevines to effectively store carbon, using a Merlot model to establish that fixed carbon within roots accounts for between 9% and 26% of the total fixed carbon within grapevines. In 15-year Cabernet Sauvignon vineyards, root carbon storage accounted for 33% of the total biomass with respect to carbon storage, and the proportion of carbon stored within roots varied from 83.7% for root systems > 6 mm (including residual roots) to just 4% for root systems <2 mm in size [19].

Studies of carbon storage to date have largely focused on soil carbon sinks in orchards [37,38,39,40], which are primarily distributed in the soil-vine interface of the soil surface in vineyards [41]. Soil organic carbon can be sequestered in the soil carbon pool for extended periods of time, potentially offsetting rising atmospheric levels of CO_2_ [42,43]. Such reports are consistent with our findings, as we determined that soil carbon storage was primarily concentrated in the surface soil (Table 3). The reason is the increased carbon input from the application of organic manure, and from litter such as branches and leaves during the vineyard management. In a 15-year-old vineyard in the Marlborough region of New Zealand, the soil carbon stores at a depth of 0–0.15 m were approximately 12 ± 5 t·hm^−2^ [44], in line with our data (Table 3). Our results highlight a practical approach to analyzing both aboveground and belowground carbon storage in vineyards, including both annual and perennial structures. Such analyses offer a convenient approach to estimating fixed carbon levels across a range of scales from the individual grape or vine to entire vineyards. They can even be adapted to evaluate entire regions or mixed crop systems. We believe that the estimates corresponding to the belowground root system and soil carbon storage discussed herein will aid future efforts to better understand such belowground carbon reserves at the vineyard level.

The present study was designed in light of several prior reports. Earlier studies largely focused upon basic grapevine physiology and development [45,46] or the carbon footprints associated with vineyard and wine production [10,47]. These prior vineyard-level analyses of carbon content primarily relied upon general estimates of the absolute carbon storage capacity of vines of different ages, together with consideration of the relative contribution of natural vegetation (vines and woody biomass) in mixed landscapes. These studies offer a more complete overview of grapevine and carbon measurement approaches based on aboveground and belowground carbon estimates, such that these methods and analytical tools can be employed to accurately estimate carbon storage at any level from an individual vine to an overall vineyard. Herein, we employed a modeling approach to quantify vine biomass and to calculate vineyard carbon storage. We further found that red grapes of Eurasian species were associated with higher biomass levels relative to similarly aged white grapes of Eurasian species (Figure 1), indicating that vineyards growing red grape varieties exhibit a higher carbon storage capacity than do those growing white grape varieties (Table 4). We further found that soil active organic carbon fractions (MBC and DOC) were mainly concentrated in the surface soil of the grapevine rhizosphere (Figure 3), indirectly suggesting that soil microorganisms are present primarily within this surface soil layer where most nutrient conversion and circulation is likely to occur, consistent with previous reports for other soil types [48,49,50,51].

With further advances in economically relevant carbon accounting strategies for vineyards, these practices are likely to become increasingly critical to informed viticultural management and decision-making efforts [52,53]. The accurate measurement of larger vineyards in this study has the potential to aid growers and local or national regulatory bodies in their ecological supervision efforts. Presently, over 38,000 ha of land is allocated for grapevine growing in the production region at the eastern foot of Helan Mountain in Ningxia. As the precise numbers of vineyards and corresponding productivity statistics are largely known, further enhancements in the accuracy of vineyard-level measurements have the potential to benefit individual farmers throughout the entire planting region, while also aiding government-led environmental protection and supervision efforts.

## 4. Materials and Methods

### 4.1. Study Site

**Figure 5:**
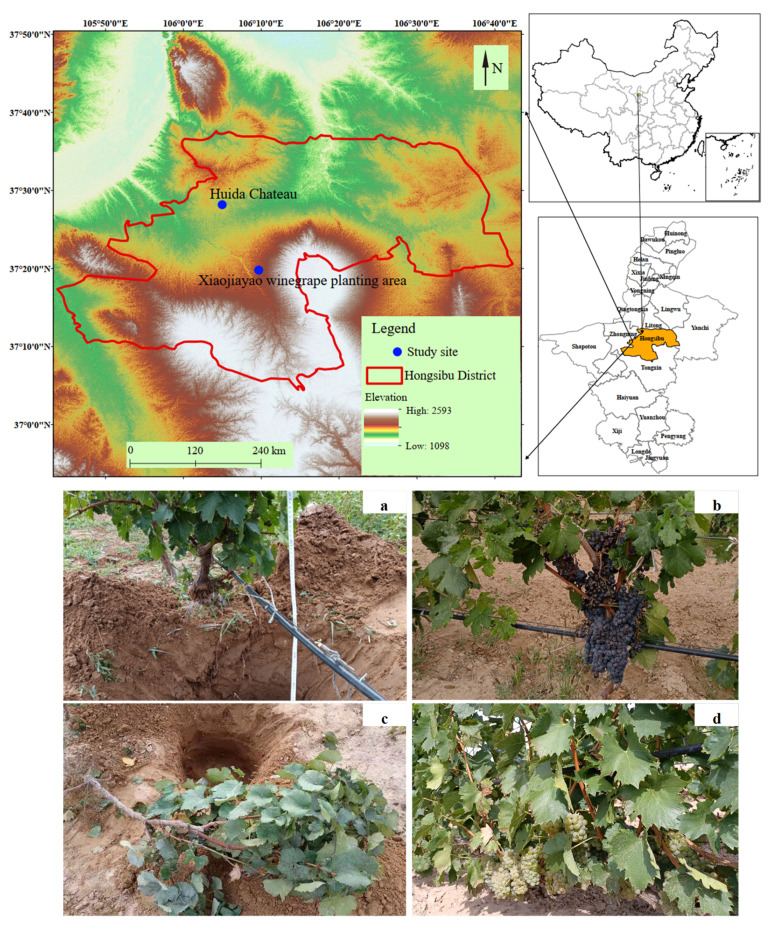
Location of the study site and the four varietals within the study area. (**a**) Cabernet Sauvignon, (**b**) Merlot, (**c**) Chardonnay, (**d**) Italian Riesling. Cabernet Sauvignon, Merlot and Chardonnay samples were collected from Huida Chateau, while Italian Riesling samples were taken from the Xiaojiayao winegrape planting area.

The present study was conducted in the Huida Chateau and Xiaojiayao winegrape planting area in the Hongsibu District of Northwest China in September 2019 (Figure 5). The study site was located within a mountain basin with an area of 2767 km^2^ at an altitude of 1240–1450 m with a typical temperate continental climate. This region has an annual average precipitation level of 251 mm, 2387 mm of average annual evaporation, an average annual temperature of 8.7 °C, a daily temperature difference of 13.7 °C, a sum of accumulated effective temperatures (≥10 °C) above 3200 °C, 2900–3550 h of annual sunshine, and an average annual wind speed of 2.9–3.7 m/s. The soil in this region exhibits a sandy loam texture (50% clay, 30% silt, and 20% sand).

Four winegrape cultivars were selected, all of which are Eurasian species (*Vitis vinifera* L.), namely the red Cabernet Sauvignon and Merlot varieties, and the white Chardonnay and Italian Riesling varieties. The Cabernet Sauvignon and Italian Riesling vines were planted in March 2012, while the Merlot and Chardonnay vines were planted in March 2017. All grapevines were regularly managed, with plants being arranged in north-south rows with a row spacing of 3 m, a vine spacing of 0.5 m, in a single-cane “Dulonggan” cultivation approach, with the exception of Italian Riesling grapes which were cultivated via a horizontal cordon training approach. Annual fertilization amounts include an estimated 40.18 kg/hm^2^ of nitrogen fertilizer, 30.21 kg/hm^2^ of phosphate fertilizer, 8.68 kg/hm^2^ of potassium fertilizer, and 15.18 t/hm^2^ of organic manure, while the annual irrigation water volume being 3900 m^3^/hm^2^.

### 4.2. Vine and Soil Sample Collection

**Figure 6:**
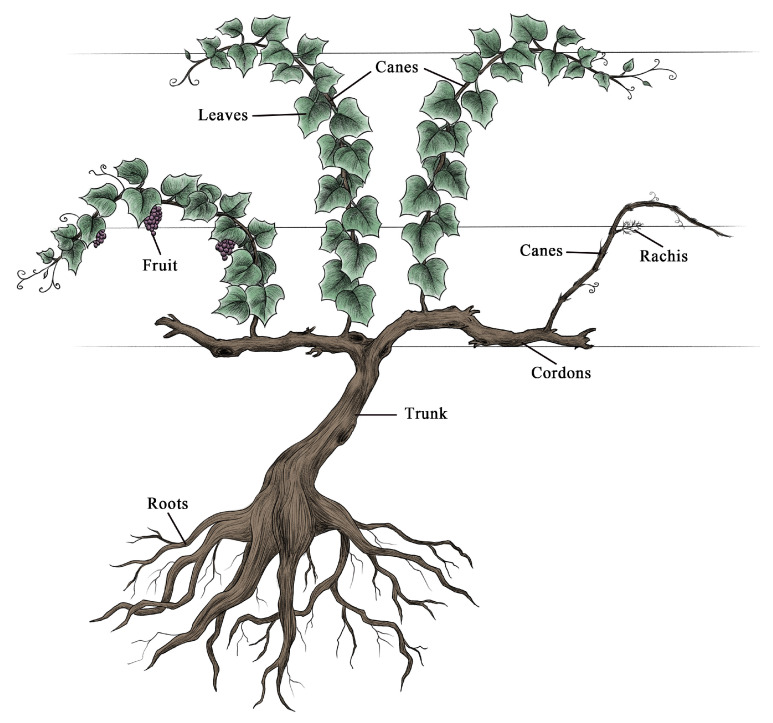
A simulation diagram for each winegrape organ. Perennial branches include both the trunk and cordons.

Four vineyard test plots of a similar size of 667 m^2^ (about 25.82 m × 25.82 m) were selected, with a planting density of 6667 vines/hm^2^, and trunk base diameter values were measured in each test plot. As the vines were very short with many branches, such that the main branch was often non-obvious, trunk base diameter was measured as an alternative to the diameter at breast height. Owing to the necessary destructive sampling techniques used herein, we selected standard grapevines to meet appropriate research needs [22]. Diameter values for vines varied in the studied vineyards, with base diameters ranging from 0.75–3.80 cm. As such, vines were grouped into 6 diameter class ranges (0.75–1.25 cm, 1.26–1.76 cm, 1.77–2.27 cm, 2.28–2.78 cm, 2.79–3.29 cm, and 3.30–3.80 cm), with 0.5 cm as the diameter used for such class sampling. In total, 30 grapevines of different diameter classes were selected to determine the biomass and carbon content therein, including 6 standard trunk base diameter values (1.0, 1.5, 2.0, 2.5, 3.0, and 3.5 cm). After harvesting these 30 vines, we divided each standard vine into aboveground (leaves, fruit, canes, and perennial branches) and belowground roots (Figure 6), after which fresh weight, dry weight, and carbon content were measured. The fruitpulp, skin, seed, and rachis components were then collected to measure the overall fruit carbon content. After the fruits were freeze-dried for 24 h, the seeds and skin were separated from the pulp. The rachis was oven-dried at 85 °C for 48 h. Each of these components was then separately ground and evenly mixed to measure fruit carbon content.

A whole root extraction method was employed for the underground roots. Owing to the relatively young ages of these vines, the excavation diameter was 0.5 m, with a depth of 1 m. Following excavation, the fresh weight, dry weight, carbon content, MBC, and DOC of each grape root sample was established. A total of 10 sampling points were selected in the vine rhizosphere of each vineyard. Four soil layers were collected (0–20 cm, 20–40 cm, 40–60 cm, and 60–100 cm) and used to assess soil bulk density and water content. Litter was removed from these soil samples, after which soil organic carbon, MBC, and DOC were measured.

### 4.3. Determination of Organic Carbon Content and Carbon Fractions

The organic carbon content of vines and soil were all heated by the Potassium dichromate external heating method [54]. MBC was extracted via chloroform fumigation and K_2_SO_4_ extraction [55]. Specifically, fumigated and unfumigated samples were extracted for 30 min with 0.5 mol/L K_2_SO_4_, with carbon concentrations in these extracts being measured via TOC and used to calculate MBC content. After collection, soil samples were passed through a 0.149 mm sieve, mixed with water at a 1:5 soil:water ratio, agitated for 30 min at 220 rpm, centrifuged at 2000 rpm for 20 min, then passed through a 0.45 μm water filter, with carbon concentrations in these extracts being measured via TOC and used to calculate the DOC content.

### 4.4. Biomass Model Selection

Standard biomass models include linear, nonlinear, and polynomial models, of which nonlinear models are most frequently utilized [56,57,58]. The allometric model is the most representative nonlinear model, as it employs a power function relationship to reflect the proportional and coordinated growth of the various components within a given ecosystem. As it incorporates easily measured parameters such as diameter at breast height and has a simple structure, stable parameter estimates, and strong predictive utility [59], this allometric model has been widely used in biomass estimation studies [60,61]. We therefore selected an allometric model to assess grapevine biomass in the present study based upon measuring the trunk base diameter and the biomass of each organ on a standard vine using the following formula:

Y = aX^b^ (1)

where X represents the trunk base diameter, and Y represents the organ biomass, while a and b are constants obtained from the regression of each organ of the standard vine.

### 4.5. Carbon Storage Estimation

Carbon storage within a vineyard was defined as the sum of grapevine biomass carbon storage and soil carbon storage. The average base diameter of each diameter class in the biomass model for each organ was used to calculate the corresponding organ biomass values, which were then multiplied by the corresponding planting density (6667 vines/hm^2^) to determine the organ biomass of each diameter class per unit area. The organ biomass carbon storage for each diameter class was defined as the organ biomass of each diameter class multiplied by the organ carbon content, with the sum of the biomass carbon storage levels for each diameter class being representative of the overall grapevine biomass within a given vineyard.

Soil carbon storage in layers to a depth of 100 cm, excluding surface litter, was calculated as follows:

(2) $$S_{d}=\sum_{i=1}^{d}D_{i}C_{i}H_{i}$$

where *S*_d_ is the soil carbon storage per unit area within a soil layer of depth d, *D_i_* represents the bulk storage of the *i*-th soil layer, *C_i_* represents the carbon content of the *i*-th soil layer, and *H_i_* represents the depth of the *i*-th soil layer.

### 4.6. Data Analysis

Carbon content and carbon fractions in different soil layers and trunk base diameter classes were compared via one-way analyses of variance (ANOVAs). Shapiro–Wilk and Levene’s tests were used to assess the normality and homogeneity of variance for data distributions, respectively. Data were then compared via one-way ANOVAs, with Fisher’s least significant difference (LSD) test and Duncan analyses being used for multiple comparisons. Correlation and linear regression analyses of normally distributed data were performed using SPSS v 24.0, with *p* < 0.05 as the threshold of statistical significance.

## 5. Conclusions

Wine remains an important commodity throughout the world, and viticulture can have a major impact on local economies. Much like orchards and plantations, grapevines are a perennial crop that can store carbon in woody tissues, thereby mitigating the emission of greenhouse gases. In the present study, we were able to generate reliable estimates of grapevine carbon storage capacities and we successfully developed tools that can easily be used by growers to estimate carbon storage in both grapevines and vineyards as a whole. This allometric model-based equation can estimate biomass-related storage in a scalable manner, and further refinement of this model and underlying agricultural management practices may lead to the recognition of vineyards and other perennial woody crops as valuable carbon sinks. We also conducted an accurate analysis of the carbon storage potential of soil and measured soil organic fractions within the grapevine rhizosphere. Overall, the carbon distribution analyses conducted herein offer a novel, detailed method of estimating the properties of such carbon sinks. If this method is widely used across a variety of planting systems, then associated measurement accuracy will continue to improve such that the spatiotemporal patterns of carbon distribution within vineyards can be more readily understood.

## Figures and Tables

**Table 1:** Allometric model-based biomass of grapevine organs.

Vines	Allometrics	Leaves	Fruit	Canes	Perennial Branches	Roots
Cabernet Sauvignon	Model	Y = 0.3489 X^0.5591^	Y = 0.3623 X^0.5502^	Y = 0.3116 X^0.6328^	Y = 0.3297 X^0.8481^	Y = 0.4302 X^0.9489^
	Correlation coefficient	0.8544 **	0.8439 **	0.8609 **	0.8910 **	0.9162 **
Merlot	Model	Y = 0.0924 X^1.2318^	Y = 0.1001 X^1.2208^	Y = 0.0873 X^1.3182^	Y = 0.1105 X^1.5151^	Y = 0.1847 X^1.4888^
	Correlation coefficient	0.9225 **	0.8989 **	0.8977 **	0.9177 **	0.9448 **
Chardonnay	Model	Y = 0.0442 X^1.5683^	Y = 0.0550 X^1.4929^	Y = 0.0515 X^1.4353^	Y = 0.0671 X^1.6698^	Y = 0.1029 X^1.6209^
	Correlation coefficient	0.9398 **	0.8992 **	0.8980 **	0.9031 **	0.9019 **
Italian Riesling	Model	Y = 0.2342 X^0.7906^	Y = 0.2432 X^0.8090^	Y = 0.2466 X^0.7526^	Y = 0.2504 X^0.9841^	Y = 0.3585 X^0.8659^
	Correlation coefficient	0.9400 **	0.9157 **	0.9295 **	0.9075 **	0.8314 **

Notes: ** *p* < 0.01.

**Table 2:** The carbon content of each organ in standard vines.

Vines	Trunk Base Diameter (cm)	Leaves (g/kg)	Fruit (g/kg)	Canes (g/kg)	Perennial Branches (g/kg)	Roots (g/kg)	*F* Value	*p* Value
Cabernet Sauvignon	1.0	410.45 ± 0.92 e	475.21 ± 1.43 a	429.46 ± 0.80 d	459.34 ± 0.72 b	451.89 ± 3.82 c	523.45	<0.01
	1.5	423.17 ± 3.07 d	478.50 ± 1.56 a	432.55 ± 2.28 c	460.85 ± 1.66 b	457.55 ± 6.26 b	127.75	<0.01
	2.0	426.87 ± 1.74 e	481.27 ± 0.70 a	435.10 ± 1.40 d	461.78 ± 1.05 b	459.23 ± 0.91 c	970.51	<0.01
	2.5	430.21 ± 1.52 e	486.19 ± 0.46 a	437.78 ± 1.52 d	468.19 ± 2.63 b	461.13 ± 1.02 c	609.86	<0.01
	3.0	432.01 ± 3.25 e	489.06 ± 2.17 a	440.31 ± 0.89 d	469.98 ± 1.34 b	461.13 ± 1.03 c	394.80	<0.01
	3.5	435.13 ± 2.26 e	496.28 ± 2.88 a	447.79 ± 2.00 d	475.32 ± 3.28 b	461.13 ± 1.04 c	268.95	<0.01
Merlot	1.0	399.19 ± 10.91 c	454.15 ± 20.32 a	409.56 ± 9.36 bc	438.61 ± 19.28 a	430.57 ± 12.33 ab	6.43	<0.01
	1.5	403.61 ± 13.03 c	459.02 ± 21.18 a	412.71 ± 10.85 bc	440.93 ± 21.37 ab	431.84 ± 18.22 abc	4.82	<0.05
	2.0	407.05 ± 11.22 c	459.43 ± 20.24 a	414.45 ± 10.34 bc	442.84 ± 22.22 ab	434.39 ± 18.15 abc	4.62	<0.05
	2.5	409.41 ± 7.80 c	462.73 ± 21.78 a	417.79 ± 9.44 bc	445.41 ± 20.96 ab	438.07 ± 21.38 abc	4.53	<0.05
	3.0	410.96 ± 14.06 c	466.45 ± 20.69 a	420.81 ± 11.70 bc	446.67 ± 20.20 ab	439.56 ± 20.98 abc	4.44	<0.05
	3.5	415.23 ± 1042 c	470.50 ± 20.82 a	423.61 ± 17.14 bc	449.54 ± 18.52 ab	447.07 ± 18.34 abc	4.82	<0.05
Chardonnay	1.0	399.01 ± 10.83 c	454.32 ± 19.85 a	408.38 ± 8.28 bc	439.66 ± 19.29 ab	431.80 ± 22.84 ab	5.24	<0.05
	1.5	403.41 ± 11.02 c	459.82 ± 24.02 a	411.88 ± 7.91 bc	441.62 ± 20.97 ab	433.61 ± 15.99 abc	5.34	<0.05
	2.0	406.39 ± 12.56 c	461.70 ± 21.92 a	415.78 ± 9.57 bc	443.33 ± 20.18 ab	436.59 ± 19.22 abc	4.85	<0.05
	2.5	410.60 ± 9.10 c	463.95 ± 20.05 a	417.22 ± 9.61 c	445.24 ± 21.99 ab	438.03 ± 22.81 ab	4.42	<0.05
	3.0	411.84 ± 13.17 c	469.15 ± 20.96 a	420.55 ± 8.21 bc	446.80 ± 21.35 ab	440.87 ± 20.54 abc	4.92	<0.05
	3.5	415.11 ± 13.38 c	471.21 ± 20.41 a	424.00 ± 7.64 bc	448.67 ± 17.71 ab	448.78 ± 17.98 ab	5.78	<0.05
Italian Riesling	1.0	395.69 ± 6.06 e	453.27 ± 4.69 a	407.45 ± 1.20 d	437.99 ± 1.80 b	430.60 ± 3.31 c	109.33	<0.01
	1.5	401.74 ± 0.58 e	458.99 ± 0.41 a	408.50 ± 2.74 d	442.48 ± 2.83 b	434.28 ± 2.77 c	359.73	<0.01
	2.0	407.36 ± 2.35 e	464.77 ± 3.41 a	413.83 ± 1.81 d	444.52 ± 4.58 b	437.26 ± 4.49 c	133.15	<0.01
	2.5	408.13 ± 2.34 d	464.52 ± 3.99 a	420.20 ± 2.77 c	446.31 ± 6.22 b	440.76 ± 5.58 b	74.67	<0.01
	3.0	411.84 ± 3.30 e	469.50 ± 3.04 a	418.65 ± 3.05 d	448.11 ± 2.50 b	441.33 ± 3.91 c	159.05	<0.01
	3.5	413.35 ± 3.28 d	476.42 ± 4.07 a	425.29 ± 4.33 c	452.45 ± 2.95 b	455.14 ± 4.44 b	127.68	<0.01

Note: The letters after the data indicate significance of the difference of multiple comparisons. Data are means ± standard deviation (SD), *n* = 3.

**Table 3:** Bulk density, carbon content, and carbon storage in each soil layer in vineyards.

Vineyards	Soil Layer (cm)	Bulk Density (g/cm^3^)	Carbon Content (g/kg)	Carbon Storage (t·hm^−2^)	Carbon Storage as a Percentage (%)
Cabernet Sauvignon	0–20	1.10 ± 0.04 d	6.81 ± 0.69 a	15.03 ± 1.47 a	34.87
	20–40	1.20 ± 0.05 c	4.72 ± 0.82 b	11.35 ± 2.14 b	26.32
	40–60	1.27 ± 0.05 b	3.03 ± 0.51 c	7.74 ± 1.50 c	17.96
	60–100	1.33 ± 0.05 a	1.68 ± 0.55 d	8.99 ± 3.20 c	20.86
Merlot	0–20	1.08 ± 0.04 d	5.82 ± 0.55 a	12.56 ± 1.28 a	30.95
	20–40	1.18 ± 0.03 c	4.59 ± 0.62 b	10.83 ± 1.41 b	26.68
	40–60	1.24 ± 0.04 b	3.55 ± 0.50 c	8.78 ± 1.26 c	21.63
	60–100	1.34 ± 0.04 a	1.56 ± 0.43 d	8.41 ± 2.47 c	20.72
Chardonnay	0–20	1.04 ± 0.03 d	5.70 ± 0.49 a	11.86 ± 1.13 a	32.31
	20–40	1.16 ± 0.06 c	4.39 ± 0.97 b	10.16 ± 2.27 b	27.67
	40–60	1.24 ± 0.06 b	3.01 ± 0.96 c	7.47 ± 1.76 c	20.35
	60–100	1.32 ± 0.08 a	1.38 ± 0.38 d	7.22 ± 1.84 c	19.67
Italian Riesling	0–20	1.06 ± 0.04 a	5.83 ± 0.64 a	12.38 ± 1.36 a	35.69
	20–40	1.15 ± 0.06 a	3.62 ± 0.52 b	8.37 ± 1.42 b	24.13
	40–60	1.34 ± 0.23 b	2.64 ± 0.53 c	7.20 ± 2.62 b	20.75
	60–100	1.34 ± 0.04 b	1.26 ± 0.29 d	6.74 ± 1.50 b	19.43

Note: The letters after the data indicate significance of the difference of multiple comparisons. Data are means ± standard deviation (SD), *n* = 10.

**Table 4:** Carbon storage distribution of vineyard ecosystems.

Vineyards	Vines		Soil		Total Carbon Storage (t·hm^−2^)
	Carbon Storage (t·hm^−2^)	Percentage (%)	Carbon Storage (t·hm^−2^)	Percentage (%)	
Cabernet Sauvignon	12.23	22.10%	43.12	77.90%	55.35
Merlot	4.41	9.80%	40.58	90.20%	45.00
Chardonnay	2.77	7.02%	36.71	92.98%	39.49
Italian Riesling	9.89	22.19%	34.69	77.81%	44.59

## Data Availability

Data is contained within the article or Supplementary Materials.

## Supplementary Materials

The following are available online at https://www.mdpi.com/article/10.3390/plants10061199/s1, Table S1: The total biomass, carbon content, and carbon storage of each winegrape organ.
