# Supplementary material for: Carbon Storage Distribution Characteristics of Vineyard Ecosystems in Hongsibu, Ningxia
Source: Plants (Basel). 2021 Jun 11;10(6):1199. doi: 10.3390/plants10061199 (PMC8231109; doi:10.3390/plants10061199)
Supplement: Supplementary file 1 [file plants-10-01199-s001.zip › plants-1246875-supplementary.pdf]

Table S1. The total biomass, carbon content, and carbon storage of each winegrape organ

Vineyards	Organ	Total Biomass of Each Organ (kg·hm^{-2})	Carbon Content (g/kg)	Carbon Storage (t·hm^{-2})	Carbon Storage as a Percentage (%)
Cabernet Sauvignon	Leaves	4279.75	426.31	1.85	15.12%
	Fruit	4400.81	484.42	2.16	17.63%
	Canes	4145.50	437.17	1.83	14.97%
	Perennial branches	5566.02	465.91	2.62	21.42%
	Roots	8123.46	460.57	3.78	30.87%
Merlot	Leaves	1450.18	407.57	0.59	13.39%
	Fruit	1558.09	462.05	0.72	16.29%
	Canes	1462.68	416.49	0.61	13.79%
	Perennial branches	2154.07	444.00	0.96	21.67%
	Roots	3527.72	436.92	1.54	34.87%
Chardonnay	Leaves	871.81	407.73	0.36	12.82%
	Fruit	1024.31	463.36	0.47	17.11%
	Canes	918.28	416.30	0.38	13.79%
	Perennial branches	1430.92	444.22	0.64	22.92%
	Roots	2113.16	438.28	0.93	33.37%
Italian Riesling	Leaves	3706.62	406.35	1.52	15.41%
	Fruit	3928.29	464.58	1.85	18.67%
	Canes	3742.16	415.65	1.57	15.90%
	Perennial branches	4912.45	445.31	2.20	22.29%
	Roots	6167.67	439.89	2.74	27.73%
